# Irony and Perspective-Taking in Children: The Roles of Norm Violations and Tone of Voice

**DOI:** 10.3389/fpsyg.2021.624604

**Published:** 2021-06-03

**Authors:** Franziska Köder, Ingrid Lossius Falkum

**Affiliations:** ^1^Department of Linguistics and Scandinavian Studies, Center for Multilingualism in Society Across the Lifespan, University of Oslo, Oslo, Norway; ^2^Department of Philosophy, Classics, History of Arts and Ideas, University of Oslo, Oslo, Norway; ^3^Department of Linguistics and Scandinavian Studies, University of Oslo, Oslo, Norway

**Keywords:** irony, development, pragmatics, relevance theory, tone of voice, norms

## Abstract

In order to understand most, if not any communicative act, the listener needs to make inferences about what the speaker intends to convey. This perspective-taking process is especially challenging in the case of nonliteral uses of language such as verbal irony (e.g., “Thanks for your help!” uttered to someone who has not provided the expected support). Children have been shown to have difficulties with the comprehension of irony well into the school years, but the factors that hamper or facilitate children’s perspective-taking in irony comprehension are not well understood. This study takes as its starting point the relevance-theoretic *echoic* analysis of verbal irony, and focuses on two of irony’s distinctive features as defined by this theory: (i) the normative bias and (ii) the characteristic tone of voice. In this study, we investigated the comprehension of irony in children aged 3–8. We manipulated these two factors, namely, the violation of different types of norms and the use of different tones of voice – to see how they affected children’s processing and interpretation of irony. Using an irony comprehension task that combined picture selection and eye-tracking, we found that the type of norm violation affected 4-to 5-year-olds’ offline understanding of irony, with a better performance on moral compared with social norm violations. Tone of voice had an effect on gaze behavior in adults, but not children, although a parodic, pretense-oriented tone of voice tended to lead to more looks to the angry compared with the happy emoticon at the offset of the ironical utterance, potentially facilitating children’s irony understanding. Our results show that the understanding of irony can be detected on explicit measures around age 6 – with the emergence of second-order perspective-taking abilities – but that a sensitivity to some of irony’s features can be detected several years earlier. Finally, our study provides a novel input to the debate on the existence of a so-called literal stage in pragmatic development, in particular regarding 3-year-olds’ differential performance on the offline and online measures of irony understanding, suggesting that they are not naively mistaking ironical utterances for “ordinary” literal ones.

## Introduction

### What Is Irony?

Although children’s pragmatic abilities develop early, many studies have shown that until quite late in development, children have difficulties with so-called Gricean pragmatic inferences, which require them to go beyond the literal meaning of the linguistic form used to obtain the meaning intended by the speaker, as in implicature (e.g., Barner et al., 2011) and uses of figurative language (e.g., Winner, 1988/1997). This perspective-taking process is especially challenging in the case of verbal irony (1), where children have been shown to have difficulties well into the school years (Winner, 1988/1997; Creusere, 1999; Filippova and Astington, 2010; Glenwright and Pexman, 2010):

1. “Thanks for your help!” [*Uttered when someone has not provided the expected support*].

But the age of acquisition differs between studies, depending on the material and measures used to assess children’s comprehension. While some studies have found only poor comprehension of irony in 8-year-old children (Massaro et al., 2013; Nicholson et al., 2013) or even 13-year-olds (Demorest et al., 1984), the lower age limit for irony understanding in experimental contexts appears to be around age 6 (Dews et al., 1996; Loukusa and Leinonen, 2008; Glenwright and Pexman, 2010). Some studies have even suggested that initial signs of irony comprehension may already be present in 3- and 4-year-old children (Loukusa and Leinonen, 2008; Reccia et al., 2010). However, the factors that hamper or facilitate children’s perspective-taking in irony comprehension are not well understood (Falkum and Köder, 2020).

One proposed explanation for the relatively late development of irony comprehension is that unlike other types of nonliteral uses, such as metaphor, irony requires rather sophisticated mind-reading abilities, specifically the ability to understand second-order belief (Perner and Wimmer, 1985; Happé, 1993). Some studies have found developmental evidence of a positive correlation between the second-order theory of mind abilities and irony understanding (Happé, 1993; Massaro et al., 2013; but see Massaro et al., 2014), a finding that supports theoretical accounts of irony, which claim that it involves a speaker having a thought about a thought, more specifically dissociating herself from a thought attributed to someone else (Wilson and Sperber, 2012). However, links between theories of irony processing and developmental studies have been largely absent (Creusere, 1999; Wilson, 2013). Few developmental studies of irony have specifically tested claims made by theories of adults’ understanding of verbal irony (but see, e.g., Keenan and Quigley, 1999). Moreover, a wide variety of operational definitions are used in the developmental literature, where irony is often understood in a very broad sense to include, for example, hyperbole, understatement, satire/parody, and rhetorical questions (see, e.g., Reccia et al., 2010). But is not clear that these phenomena all involve the same mechanism, and consequently, whether they should all be expected to follow the same developmental trajectory. Needless to say, how irony is conceptualized greatly influences hypotheses about the pragmatic and cognitive abilities required for its mastery, and about the underlying causes of children’s apparent difficulties with it.

The aims of this study are as follows. We take as our starting point the relevance-theoretic *echoic* analysis of verbal irony (Wilson and Sperber, 2012), and focus on two of irony’s distinctive features as defined by this theory, regarding (i) the normative bias involved in irony and (ii) the ironical tone of voice. Based on developmental data from children aged 3–8, we investigate how these two factors – the violation of different types of norms and the use of different tones of voice – affect children’s processing and interpretation of irony. Finally, our study aims to address a general topic in the study of pragmatic development regarding whether children go through a so-called literal stage in figurative language acquisition.

### Echoic Irony: Three Distinctive Features

Relevance theory (Sperber and Wilson, 1986/1995; Wilson and Sperber, 2004) is a theory of human communication that seeks to provide a psychologically realistic explanation of how hearers infer speaker meanings by combining contextual information with the linguistic evidence provided. The central claim is that utterances – including those that involve nonliteral uses of language – create expectations of relevance, which are precise and predictable enough to guide the addressee toward the speaker’s meaning (see Wilson and Sperber, 2004; p. 608, for more detail). Relevance theory offers an alternative to the classical and Gricean views (Grice, 1975/1989) of irony as involving a deviation from a norm of literalness, which have been increasingly questioned (Gibbs, 1994; Glucksberg, 2001; Wilson and Sperber, 2012). Instead, the idea is that in verbal irony, the speaker is tacitly *echoing* a thought (a belief, an intention, a norm-based expectation) she attributes to another source (or to herself at a different time) and expressing a dismissive attitude to that thought. Thus, when Mary says “Thanks for your help” to someone who has not provided the expected support, she is expressing a dismissive attitude to the norm-based expectation that people should in general be helpful and supportive of each other. In this way, irony involves a “thought about a thought,” that is, a second-order metarepresentation, and in order to grasp it, the hearer must be able (a) to understand the utterance as echoic and (b) to recognize the speaker’s dismissive attitude to the attributed thought. Thus, the process of irony understanding places rather heavy demands on the child’s perspective-taking abilities.

Wilson and Sperber (2012) discuss three distinctive features of irony that a theory of irony must explain: (a) the characteristic dismissive (e.g., mocking, skeptical, or contemptuous) attitude it expresses; (b) the normative bias it involves, usually pointing out that a state of affairs does not live up to some norm-based expectation; and (c) the ironical tone of voice, characterized by a flat or deadpan intonation. Further, they show how each of these features follows from their account of irony as an *echoic* use of language: first, irony is directly targeted at attributed thoughts and its purpose is to convey an attitude to those thoughts (a). Second, norms, in the sense of socially shared ideas about how things should be, are always available to be ironically echoed when they are not satisfied (b). Third, the ironical tone of voice provides a cue to the particular type of dismissive attitude that the speaker intends to convey to the thought being echoed (c). When does the ability to understand irony, involving the three distinctive features in (a–c) above, emerge in development?

### Echo or Pretense?

The main contemporary competitors to the echoic account of irony developed within relevance theory are so-called pretense theories (e.g., Clark and Gerrig, 1990; Kumon-Nakamura et al., 1995). Pretense accounts see the speaker of an ironical utterance as *pretending* to perform a speech act – in (2) below this would be an assertion – in order to express a dismissive attitude to the speech act itself, or anyone who would perform it or take it seriously.

2. A [*to a particularly clumsy person*]: you’re so graceful!

A key issue in the literature is thus whether or not irony necessarily involves an element of pretense. Wilson and Sperber (2012) argue that echoing and pretense are distinct mechanisms; while all ironic utterances are echoic, only some of them involve pretense. Consider the ironical utterances in (3) below.

3. *John has just spilled a glass of wine on their new, white carpet*.
  - Mary [*with a deadpan intonation*]: oh, that’s great.
  - Mary [with an exaggerated imitation of an enthusiastic tone of voice]: oh, that’s GREAT!

While (3a) could be seen as a case of purely echoic irony, where the speaker is tacitly echoing an attributed thought and expressing a dismissive attitude to it, (3b) involves both echo and pretense and could be seen as an instance of “parodic” irony, where the speaker is imitating and thereby ridiculing the sort of person who would enthusiastically make such an exclamation. This example illustrates how the echoic and pretense accounts make different predictions about the ironical tone of voice (Sperber, 1984): on the pretense account, the speaker is expected to mimic the tone of voice of the person she is imitating. On the echoic account, the ironical speaker is not expected to leave her own voice behind, but to use instead a tone of voice designed to reflect her own dismissive attitude to the thought she is echoing.

Given children’s ability to use pretense very early in development (i.e., their beginning to engage in pretending play roughly around the age of 18 months), we may hypothesize that the imitative, exaggerated tone of voice used in pretense-based forms of irony might make it easier for them to recognize that the speaker is distancing herself from the literal speech act she is performing, and thereby positively influence their understanding. If so, this would suggest that the distinct tones of voice used in the two forms of irony may be linked to different mechanisms, with “regular” irony involving echoing alone and “parodic” irony involving both echoing and pretense.

Although the role of pretense in irony is a matter of theoretical debate, irony clearly has an affinity with children’s early-emerging ability for pretense. Further, irony is seen as closely related to humor and jokes (Gibbs et al., 2014), which also develop early (Hoicka, 2014). While the mechanisms underlying irony and jokes may be distinct (Wilson, 2017), there are some common aspects that children seem to relate to from an early age. Consider, for instance, the common parental practice of “reverse psychology.” In a situation where a young child is unwilling to do something, the parent may tell the child, using a tone of voice that signals a joking attitude, *not* to perform the desired action (e.g., “Do not put on your shoes now…!”). And the child will typically disobey the parent’s instruction with delight. In such cases, the child has recognized the parent’s pretense, which signals a dissociation from the utterance’s literal content. While these are not true cases of irony according to the standard echoic account, they share some of irony’s features (characteristic tone of voice, dissociative attitude to a propositional content). Given this, we might expect some features of irony to be understandable to children younger than 6 years, which is considered to be the lower age limit for irony understanding in experimental settings.

### Is There a Literal Stage in the Development of Nonliteral Uses of Language?

Children’s early-emerging pragmatic competence is attested across a variety of studies and pragmatic tasks (see Tomasello, 2008, for an overview), including prelinguistic communication (Stephens and Matthews, 2014), word learning (Bloom, 2000), and referential communication (Matthews et al., 2006). In this light, children’s apparent difficulties with nonliteral uses of language, that is, cases where they have to go beyond the conventional senses of the words and sentences (e.g., metonymy, metaphor, and irony), is increasingly seen as a puzzling feature of their pragmatic development. Some researchers have suggested that pre- and primary school children go through a *literal stage* in figurative language development (Asch and Nerlove, 1960; Winner et al., 1976; Winner, 1988/1997; Levorato and Cacciari, 2002), characterized by a decrease in their production of figurative language, for instance, early “metaphors” (Billow, 1981) and “metonyms” (Falkum, 2019), and a bias toward literal interpretations, before a more sophisticated level of figurative language competence is attained. For instance, Levorato and Caccari (2002; p. 129) have claimed that up to about age 7, a primitive type of processing is prevalent, which involves “a piece-by-piece elaboration of linguistic input; children process language literally even when it does not make sense in the linguistic context.”

The idea of the existence of a literal stage has been criticized in connection with the growing evidence, gleaned from studies focusing on metaphor comprehension, that attests to the presence of a figurative language competence emerging as early as the preschool years (Deamer, 2013; Di Paola et al., 2020; Pouscoulous and Tomasello, 2020). However, in a recent study of metonymy comprehension using an offline picture selection task, Falkum et al. (2017) found a U-shaped development, with 3-year-olds performing better than 4- and 5-year-olds, who tended to interpret metonymic uses literally (e.g., choosing a picture of a mustache instead of the man with the mustache for “*The mustache* sits down at the table”). In a replication of this study using a methodology that combined picture selection (offline) and eye-tracking (online), we observed the same U-shape in children aged 3–8 years, with performance starting to improve at age 6 (Köder and Falkum, 2020). However, results revealed a clear sensitivity to metonymic uses in the online eye-tracking data of *all* the participants, who preferred looking at the contextually appropriate metonymic referent, including those 4- to 5-year-old children who chose literal interpretations in the offline picture selection task. This suggests that there are properties of offline tasks that mask children’s understanding – and which might lead them to choose literal interpretations of nonliteral communicative intentions – and that gaze data from eye-tracking could be more revealing of their actual pragmatic processing of the utterance.

Relating this to standard irony comprehension tasks, they often involve children being asked complex comprehension questions such as “Did X really mean that *p*?” or “Why did X say that *p*?” requiring quite developed verbal reasoning abilities. Recent research on children’s irony comprehension, using more child-friendly tasks and implicit comprehension measures, suggests that children may not have a bias to access the literal interpretation first (Climie and Pexman, 2008; Whalen et al., 2020). Could the use of eye-tracking measures reveal that a sensitivity to certain types of ironical uses may emerge earlier than previously thought?

### Developmental Hypotheses

In this paper, we link our developmental study to theoretical accounts of irony understanding and test children’s processing and comprehension of irony, in the sense of the speaker tacitly dissociating herself from an *echoed* thought (cf. Wilson and Sperber, 2012). We focus on two of irony’s distinctive features, as defined by Wilson and Sperber (2012): (i) the normative bias and (ii) the ironical tone of voice. Specifically, we investigate how different tones of voice and the violation of different types of norms affect children’s processing and comprehension of irony. First, as mentioned above, irony is typically used when the speaker’s norm-based expectations have been violated (Wilson and Sperber, 2012), such as in (1) that the addressee should be more helpful. A growing body of work attests to a strong disposition in children to infer, adhere to, and enforce norms (and conventions) across disparate domains, including language (Clark, 2007), pretense games (Rakoczy, 2007), and social behavior (Göckeritz et al., 2014). Children not only learn norms from direct instruction and prohibition, but also seek norms themselves: for instance, 3-year-olds have been shown to spontaneously infer a social norm from a single observation of adult intentional behavior (Schmidt et al., 2016). Further, the developmental literature shows that children are sensitive to different types of norms, distinguishing between moral and social norms from an early age (Nucci and Turiel, 1978; Nucci and Nucci, 1982). Children have been shown to perceive violations of moral norms as more serious and deserving of punishment than violations of social norms (Smetana, 1981). However, it is unclear whether the type of norm that is violated affects children’s irony understanding, or whether children’s attested early sensitivity to norms might help them in detecting the mismatch between the context and the speaker’s utterance in irony. We hypothesize that irony is easier to understand when a moral norm compared with a social norm is violated (e.g., people violating an expectation to be helpful vs. violating an expectation to take off one’s hat inside) as the discrepancy between expectation and reality, as well as the severity of the violation, is potentially more salient.

Second, irony is also typically associated with a characteristic tone of voice: a flat or deadpan intonation, slower tempo, lower pitch level, and greater intensity (Rockwell, 2000; Cheang and Pell, 2008) are important cues to a speaker’s ironic intention. As we have seen, the two main theoretical accounts of irony, the echoic account (Wilson and Sperber, 2012) and the pretense account (Clark and Gerrig, 1990), make different predictions regarding the ironical tone of voice (Sperber, 1984): the pretense account expects the ironical speaker to mimic the tone of voice of the person she is imitating, leaving her own voice behind. The echoic account expects the speaker to use a tone of voice, which reflects her dismissive attitude to the echoed thought, and pretense elements are a possible additive, but not necessary ingredient. Given children’s early familiarity with pretense, we hypothesize that a parodic, exaggerated tone of voice (pretense) might make an ironical utterance easier to understand than a deadpan tone of voice (echo). Theoretically, our goal was to test these predictions regarding the ironical tone of voice: if their developmental trajectories differ, that is, if one emerges before the other, this would suggest that the distinct tones of voice used in the two forms of irony may be linked to different mechanisms.

Finally, our study addresses the ongoing discussion regarding the existence of a “literal stage” in pragmatic development. While we expect that young children would choose mainly literal interpretations in the offline picture selection task, we hypothesize that, given irony’s affinity with other pragmatic phenomena of which children show an early appreciation (pretense, jokes, humor), we would see some sensitivity to irony when including an online measure of eye-tracking, in line with our previous results for the comprehension of metonymy (Köder and Falkum, 2020). If so, it would suggest that young children, even if showing a literal preference on offline tasks, are not necessarily naively mistaking ironical utterances for “ordinary” literal ones.

## Materials And Methods

### Participants

We tested 195 Norwegian-speaking children between 2 and 8 years and a control group of 20 adults. Since the accuracy data revealed that the task was too demanding for 2-year-old children, we excluded them from further analysis. In addition, the data of one 7-year-old child was excluded due to experimenter error, leaving us with a total of 183 children (79 females) between 3 and 8 years (see Table 1). Written parental consent was obtained prior to the experiment. All children were tested individually in a separate room in their kindergarten or school on an SMI iView 250 RED mobile eye-tracker. They received a small reward (stickers) for participating.

**Table 1:** Participants.

Age group	Mean age	Range	Number (f/m)
3	3.5	3.0–3.9	27 (14/13)
4	4.5	4.0–4.9	28 (9/19)
5	5.5	5.0–5.9	25 (7/18)
6	6.5	6.0–6.9	36 (17/19)
7	7.5	7.0–7.9	27 (11/16)
8	8.6	8.0–8.9	40 (21/19)
Adults	30.6	21–57	20 (10/10)

### Experimental Design

**Figure 1:**
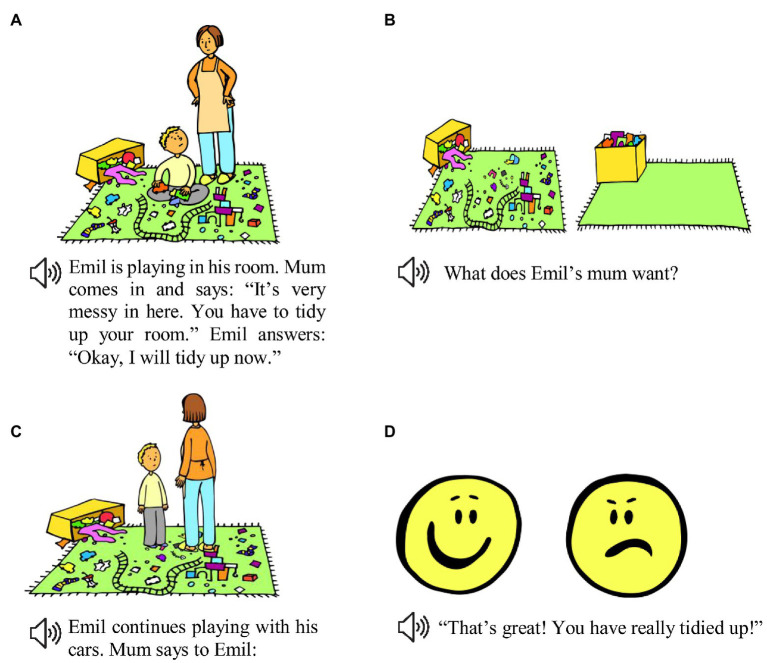
Example of an experimental story, consisting of parts **(A-D)**. Stories are presented aurally, accompanied with pictures. Participants’ gaze is measured during audio-visual presentation of **(D)**.

Participants listened to prerecorded stories, which were accompanied by pictures on a screen. This setup was chosen because of its high ecological validity, simulating children’s common experience of picture-book reading. Before presenting the experimental items, we ensured that children knew that the two emoticons used in the experiment (see Figure 1D) represented a happy or angry emotion, respectively.

All experimental stories feature interactions between a parent and a child, a constellation that has previously been shown to facilitate irony understanding in children (Massaro et al., 2013). The stories are structured in the following way: first, the parent protagonist tells the child protagonist what she expects of him. For instance, in example story (1), mum is telling Emil to tidy up his messy room (see Figure 1A). With a comprehension question (see Figure 1B), we checked whether participants understood the parent’s expectations and, in case they did not, repeated the story from the beginning. The story proceeds with the child protagonist either meeting or violating the parent protagonist’s expectations, for instance, Emil tidying up his room or leaving it in a messy state (see Figure 1C). The parent reacts by either praising or criticizing the child, using one of three types of utterances: literal praise (e.g., “That’s great! You have really tidied up”), literal criticism (e.g., “That’s bad! You have not tidied up!”), or irony (e.g., “That’s great! You have really tidied up!”) uttered with either a deadpan or parodic tone of voice.^1^ The literal or ironic target utterance starts 1,000 ms after the appearance of a happy and angry emoticon on the screen (see Figure 1D). Note that we decided to portray the story protagonists’ emotions with emoticons rather than drawings of real faces to make clear that the protagonists’ inner feelings are depicted, which are not necessarily reflected in facial expressions.

After each story, the experimenter asked the participant *How is mum/dad feeling inside? Is she/he happy* (experimenter pointing to the happy emoticon) *or angry* (experimenter pointing to the angry emoticon)? – which the participant could answer either verbally or by pointing to one of the emoticons. We counterbalanced the position of the emoticons (left, right) on the screen and in accordance also the order in which the two emotions are mentioned in the test question.

Each participant listened to 12 different stories, presented in random order. Six of them had a positive ending, that is, the child met the parent’s expectations and received praise, and six had a negative ending, that is, the child violated the parent’s expectations and was criticized either literally or ironically. All participants were randomly assigned to one of two tones of voice conditions (deadpan or parodic). Of the 12 stories, three involved moral norms (e.g., people should not hurt each other), three social norms (e.g., hands should be washed before eating), and three personal preferences (e.g., mum wants child to put on a nice dress).

### Auditory Stimuli

The auditory stimuli for the experiment were recorded in a silent room with a high-quality recording device (H2 Zoom Handy Recorder). The recorded speaker, a male native speaker of Norwegian, received extensive training beforehand so that he could produce the two distinct types of ironic tone of voice naturally and consistently. For the deadpan stimuli, he was instructed to produce an ironical utterance with a flat, monotonous intonation. For the parodic stimuli, he was instructed to pretend to be happy while simultaneously expressing a dismissive attitude. From several recordings, we selected the ones that exhibited the specific auditory characteristics of a deadpan and a parodic tone of voice most clearly. The same speaker also produced utterances for the two literal control conditions. For literal praise, he was instructed to use a sincerely happy tone of voice and for the literal criticism an angry tone of voice.

#### Acoustic Analysis

All auditory stimuli were acoustically analyzed with the PRAAT software (version 6.1.15). As Table 2 shows, the four types of utterances exhibit specific acoustic characteristics, with literal praise having, for instance, a higher fundamental frequency than literal criticism. In the statistical analysis, we focus on comparing deadpan and parodic irony, using *t*-tests with Bonferroni correction to correct for multiple comparisons. Comparing the two types of ironical tones of voice, we find that deadpan irony is uttered with lower intensity (*p* < 0.001), and its mean fundamental frequency is lower, even though not significantly different from that of parodic irony (*p* = 0.32).

**Table 2:** Acoustical analyses for different types of utterances: mean duration, mean intensity, and mean F0.

	Deadpan irony	Parodic irony	Literal praise	Literal criticism
Duration (s)	2.2 (0.4)	2.4 (0.4)	2.2 (0.3)	2.3 (0.4)
Intensity (dB)	57.0 (2.0)	66.4 (2.2)	62.9 (1.7)	58.5 (4.5)
F0 (Hz)	132.1 (52.5)	158.6 (29.4)	201.3 (47.9)	128.9 (22.3)

#### Perceptual Evaluation

To determine whether the tone of voice of our auditory stimuli is a successful cue to the speaker’s ironical intent, we conducted a perception study with 35 adult native speakers of Norwegian (mean age: 35, age range: 20–73). The participants listened to 36 recorded utterances of the type deadpan irony (*n* = 12), parodic irony (*n* = 12), and literal praise (n=12) that were presented in a random order without any contextual support. All utterance types have identical wording but differ in their acoustic properties. Participants were instructed to rate, based on the speaker’s tone of voice, whether they agreed or disagreed that the speaker is being ironical/sarcastic (1 meaning strongly agree, 5 meaning strongly disagree). The results show that both deadpan (*mean* = 2.05, *SD* = 0.92) and parodic utterances (*mean* = 2.12, *SD* = 1.09) were perceived as ironical, while literal praise utterances were not (*mean* = 4.59, *SD* = 0.68). Pairwise *t*-tests with Bonferroni adjustments indicate that utterances of the types deadpan and parodic irony differed significantly from literal praise (*p* < 0.001), but not from each other (*p* = 0.825).

## Results

### Picture Selection Results

**Figure 2:**
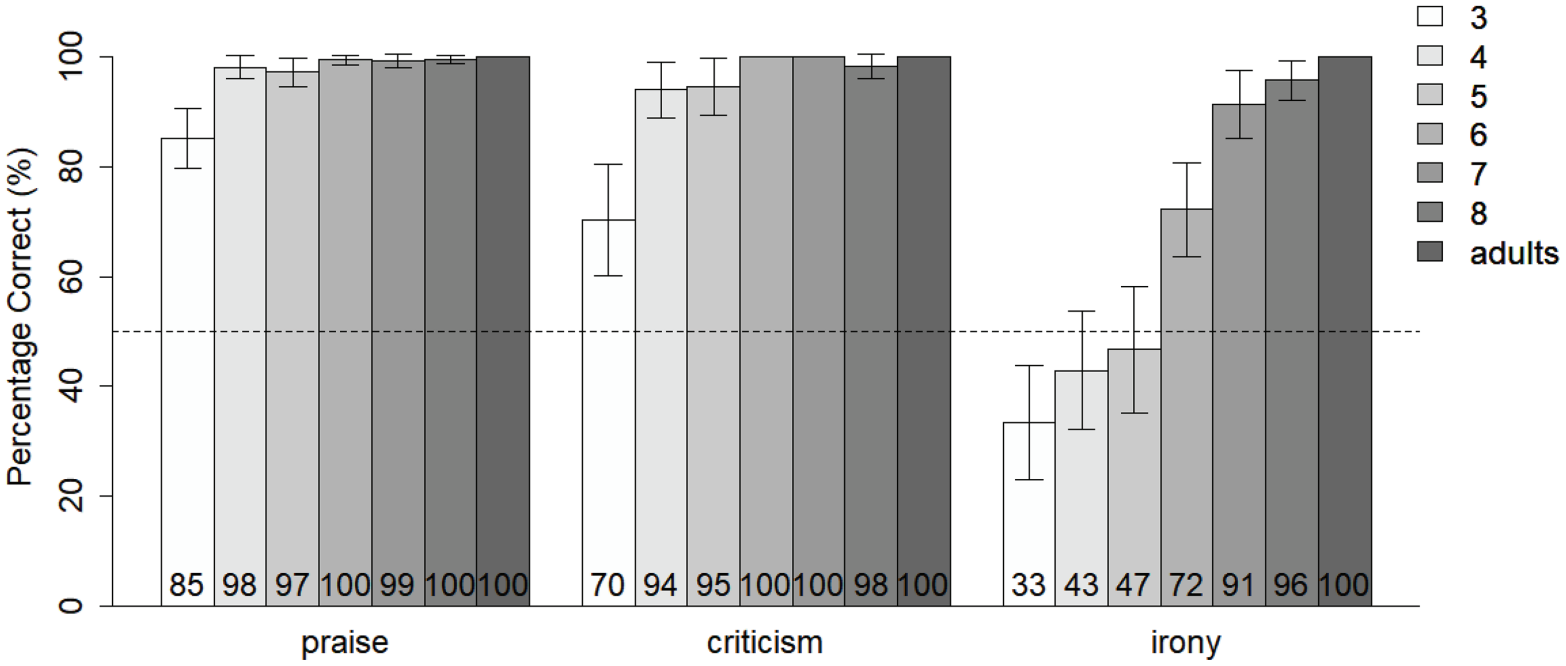
Percentage of correct picture choice for different utterance types (literal praise, literal criticism, and irony) and age groups (3–8, adults). Error bars represent 95% confidence intervals. Dotted line indicates chance level.

Figure 2 shows the results from the picture selection task, in which participants were asked to choose the emoticon (happy/angry) that matched the feeling of the story protagonist best. Participants of all age groups performed well above chance in the literal praise and literal criticism condition. In the irony condition, 3-year-olds performed below chance, showing a bias toward literal interpretations of ironical utterances, while 4- and 5-year-old children performed at chance level. From the age of 6, children were mostly able to correctly assess the emotion of the ironical speaker, with 7- and 8-year-old children approaching adult-like performance.

We analyzed participants’ accuracy of picture selection with generalized linear mixed-effects modeling (GLMM), using the “lme4 package” in the software R (version 3.6.1). Stepwise, random effects and fixed effects were added to a logistic regression model that improved the model fit significantly (decrease of AIC by more than 2; Akaike, 1974). The final model includes random intercepts for subjects and items and fixed effects for utterance type (praise, criticism, and irony) and age.^2^ Based on the model, participants were significantly more often correct in their interpretation of literal praise (*β* = 4.13; *z* = 10.17; *p* < 0.001) or literal criticism (*β* = 3.15; *z* = 7.99; *p* < 0.001) compared with irony. Age has a positive effect on accuracy (*β* = 1.04; *z* = 10.13; *p* < 0.001). The type of norms (moral, social, and personal preference) addressed in the story, the tone of voice of the ironical utterances (deadpan and parodic), and the gender of participants (f/m) did not influence participants’ picture choice. There was also no indication that participants’ performance improved or deteriorated during the course of the experiment.

In the next step, we focused on the irony comprehension of 4- to 5-year-old children, who are in a transitional phase in irony acquisition, between a possible literal bias (age 3 and younger) and more robust irony understanding (age 6 and above). With generalized linear mixed effects models, we investigated whether the factors norm type and tone of voice influence irony comprehension in 4- and 5-year-old children. Our analysis reveals that tone of voice did not influence irony understanding, but that norm type had an effect: 4- to 5-year-olds tended to understand ironical utterances better when moral norms were violated as compared with personal preferences (*β* = 1.11; *z* = 2.13; *p* = 0.03) or social norms (*β* = 0.86; *z* = 1.68; *p* = 0.09), even though the latter was not statistically significant. When we combined the categories “social norms” and “personal preferences,” we found that 4- to 5-year-olds performed better on irony understanding in stories where moral norms rather than nonmoral expectations (i.e., social norms and personal preferences) were violated (*β* = 0.98; *z* = 2.18; *p* = 0.03).^3^

### Gaze Results

We analyzed participants’ gaze to the two emoticons during the processing of the target utterances (irony, criticism, and praise), with looks to the angry emoticon compared with looks to the happy emoticon as a dependent variable. The analyzed time window spans from the onset of the target utterance, 1,000 ms after the image of the two emoticons appeared on the screen, until the average offset of the target utterance at 3,300 ms, adding 500 ms after the offset to take into account that young children need more time to initiate a saccade (Yang et al., 2002; Irving et al., 2006). We are interested in the looking behavior of three age groups, based on the accuracy analysis: younger children (3–5 years), who are still at chance level or below in their irony interpretation; older children (6–8 years), who are on average able to interpret ironical utterances correctly; and adults.

We analyzed the gaze data with generalized additive mixed modeling (GAMM) in R, using the R package “mgcv” (version 1.8–28; Wood, 2017). A GAMM analysis has the advantage that it allows for the modeling of nonlinear time-course effects, typical for gaze data in the visual world paradigm (Porretta et al., 2017). Stepwise, we included parametric and nonparametric factors, using the compareML function from the R package “itsadug” (van Rij et al., 2017) for model comparison. To investigate the interpretation of different types of utterances (irony, criticism, and praise) across different age groups (younger children, older children, and adults), we created the combined factor “Utterance type-Age group” with nine levels, such as “irony-adults” or “criticism-younger children.” Our final model, presented in Table 3, includes the parametric predictor Utterance type-Age group, a time by-Utterance type-Age group smooth, and random intercepts for items.^4^

**Table 3:** Generalized additive mixed model reporting parametric coefficients (Part A), and smooth terms and random effects (Part B).

**Part A**				
**Parametric coefficients**	**Estimate**	**Std. Error**	*z* **value**	*p* **value**
(Intercept)	0.884	0.063	13.835	<0.001
Criticism-Adults	−0.096	0.107	−0.902	0.367
Criticism-Older	−0.533	0.104	5.121	<0.001
Criticism-Younger	−0.621	0.104	−5.964	<0.001
Irony-Older	−0.690	0.033	−20.959	<0.001
Irony-Younger	−0.768	0.032	−23.685	<0.001
Praise-Adults	−2.069	0.105	−19.660	<0.001
Praise-Older	−1.281	0.104	−12.379	<0.001
Praise-Younger	−1.102	0.104	−10.636	<0.001
**Part B**				
**Smooth terms**	**edf**	**Ref.df**	**Chi.sq**	*p* **value**
s(Time): Irony-Adults	6.357	7.509	49.50	<0.001
s(Time): Criticism-Adults	7.902	8.685	54.24	<0.001
s(Time): Criticism-Older	8.169	8.812	197.14	<0.001
s(Time): Criticism-Younger	8.128	8.792	187.93	<0.001
s(Time): Irony-Older	7.284	8.284	63.63	<0.001
s(Time): Irony-Younger	8.014	8.737	87.23	<0.001
s(Time): Praise-Adults	7.120	8.163	393.43	<0.001
s(Time): Praise-Older	7.091	8.143	277.28	<0.001
s(Time): Praise-Younger	8.073	8.768	217.57	<0.001
Random intercepts for items	44.063	45.00	2120.23	<0.001

**Figure 3:**
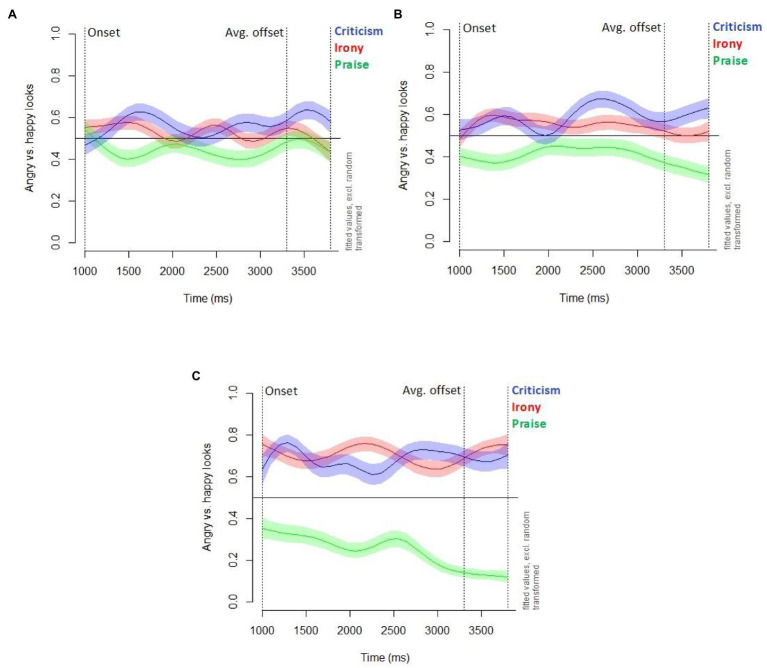
Estimated difference in proportion of looks to angry vs. happy emoticon for different utterance types (criticism, irony, praise) in **(A)** Younger children aged 3-5 years, **(B)** Older children aged 6-8 years, and **(C)** Adults. Estimations are based on the GAMM presented in Table 3.

To visualize the model, we plotted the summed effects, using the “plot_smooth” function from the R package “itsadug” (van Rij et al., 2017; see Figure 3). If the graph is above 0.5, this means that participants looked more to the angry compared with the happy emoticon; if it is below 0.5, participants looked more to the happy compared with the angry emoticon; if it is equal to 0.5, participants looked equally often to the happy and angry emoticon.

The gaze data of the adults (Figure 3C) reveal clearly distinct looking patterns for literal praise on the one hand – with more looks to the happy emoticon (graph below 0.5) – and irony and literal criticism on the other hand – with more looks to the angry emoticon (graphs above 0.5). Similar to adults, older children (Figure 3B) showed distinct looking patterns for ironical utterances compared with literal praise utterances throughout the whole time frame. However, for 6-to 8-year-olds also, the looking patterns for ironical utterances and literal criticism differed significantly: from 3,375 ms onward, they looked less at the angry emoticon when listening to ironical utterances, with an equal amount of looks to both emoticons in the irony condition from around 3,300 ms. Younger children (Figure 3A) also showed a clear tendency to look more at the angry emoticon when listening to ironical utterances compared with literal praise utterances. However, from 3,347 ms, the graphs for irony and literal praise do not differ significantly anymore. Around the same time (3,375 ms), younger children’s looking patterns in the irony and literal criticism condition start to differ from each other.^5^ Figure 3 also reveals an age-related trend: the older the participants, the bigger the difference in “angry vs. happy looks” between literal praise, on the one hand, and the two forms of criticism (irony and literal criticism), on the other hand, visible as the spatial distance between the graphs.

Next, we analyzed whether tone of voice and norm type influence participants’ looks in the irony condition, using generalized linear mixed models. For the complete time frame (1,000–3,800 ms), we did not find an effect of tone of voice or norm type for neither adults nor children. However, when focusing on the time window after the offset of the target utterance (3,300–3,800 ms), which in the GAMM analysis showed the most distinct looking patterns for the different age groups, we can notice interesting effects. Adults looked significantly more to the angry compared with the happy emoticon when irony was produced with a deadpan compared with a parodic tone of voice (*β* = 12.60; *z* = 1.96; *p* = 0.05).^6^ For children, we found a tendency toward the reverse effect, with the parodic tone of voice leading to more looks to the angry compared with the happy emoticon (*β* = 1.77; *z* = 2.05; *p* = 0.04).^7^ However, when we included random intercepts for items in the model, this effect was not significant any more (*β* = 1.83; *z* = 1.25; *p* = 0.21). The type of norm did not affect eye gaze in children and adults.

## Discussion

We investigated children’s irony processing and understanding by combining the offline measure of picture selection with the online measure of eye-tracking. The data from the picture selection task show a clear improvement of irony understanding with age: 3-year-olds were below chance in the interpretation of irony, while 4- and 5-year-old children were at chance level. From the age of 6, children have already a good understanding of verbal irony, which improves further in the years after. The fact that even the youngest children we tested, the 3-year-olds, performed well in the two literal control conditions confirms that the task was age-appropriate. The gaze data provide novel information about the processing of ironical utterances compared with utterances with the same wording (literal praise), and utterances with the same context and similar negative valence (literal criticism). In adults, we can see a two-way distinction between the processing of utterances with positive and negative valence, with no significant difference in looking behavior between irony and literal criticism. Older children, aged 6–8, exhibit a similar distinction between the processing of literal praise on the one hand and irony and literal criticism on the other hand. However, toward the end of an ironical utterance, these older children looked equally often to the happy as to the angry emoticon. This could be an indication that the literal meaning is more prominent during children’s than during adults’ processing of irony. The below-chance performance of 3-year-olds’ in the picture selection task suggests that they have a strong bias to interpret ironical utterances literally. However, the gaze data show that ironical utterances led to significantly more looks to the angry compared with the happy emoticon during most of the utterance, suggesting that 3- to 5-year-olds are sensitive to cues such as context or tone of voice, which are indications that the speaker is likely to have a negative attitude. However, once 3- to 5-year-olds have analyzed the complete utterance, the gaze pattern for ironical utterances resembles that of literal praise utterances.

### Norm Type

Although the developmental literature shows that children distinguish between moral and social norms from an early age, we found only partial support for the hypothesis that the type of norm violated influences children’s irony understanding. Interestingly, children aged 4–5, who are on the verge of understanding irony, tended to perform better in the picture selection task when a moral norm such as “do not hurt others” was violated than when a social norm (e.g., “wash your hands before dinner”) or personal preference (e.g., mother’s clothing preference) was violated. If children perceive moral norm violations as more severe and punishable than violations of social norm (Smetana, 1981; or violations of personal preferences), this might have made the discrepancy between expectation and reality more salient and thereby reduced the likelihood that the ironical utterance was meant literally. This, in turn, might have made it easier for children to notice the dismissive attitude of the ironical speaker and discard the literal interpretation. However, for children aged 6 and older, who in general performed well on irony comprehension, we did not find an effect of norm type violated. This might be because once the perspective-taking abilities necessary for irony comprehension are mastered, it does not matter so much what sort of norm violation underlies the ironical utterance. If anything, it might be slightly less appropriate to use irony in a moral norm violation setting, because the perceived severity of the violation may not lend itself naturally to the mockery that is typically involved in ironical uses. However, we did not find evidence of this in our data.

### Tone of Voice

We also investigated whether the tone of voice used with the ironical utterance influences children’s understanding, comparing a deadpan (flat, slower tempo, lower pitch level, and greater intensity) and a parodic (imitative and exaggerated) intonation. As mentioned above, the two competing accounts of irony, the *echoic* account (Wilson and Sperber, 2012) and the *pretense* account (Clark and Gerrig, 1990), make different predictions about the ironical tone of voice (Sperber, 1984): while on the pretense account, the speaker is expected to mimic the tone of voice of the person she is imitating, on the echoic account, the ironical speaker is not expected to leave her own voice behind, but to use instead a tone of voice designed to reflect her own dismissive attitude to the thought she is echoing. Our hypothesis was that the imitative, exaggerated tone of voice used in pretense-based forms of irony might make it easier for children, who are already familiar with pretend situations, to recognize that the speaker is distancing herself from the literal speech act she is performing, and thereby positively influence their understanding.

While we did find support for an effect of tone of voice in our data, this was subtler than predicted. In the gaze data, adults looked more at the angry emoticon during the final phase of the utterance when a deadpan tone of voice was used. This could indicate that for adults a deadpan tone of voice is the default tone of voice for verbal irony and therefore more strongly linked to a negative attitude, a finding that would support the echoic account of irony. By contrast, in the same time window, there was an indication for the opposite effect in children. They tended to look more at the angry emoticon when irony was uttered with a parodic tone of voice, suggesting that the imitative, exaggerated tone of voice used in pretense-based irony made it easier for them to recognize the speaker’s nonliteral intention. Such a tone of voice is already familiar to children from other types of pretense contexts, including humor, jokes, and “reverse psychology,” which share some of irony’s attributes (e.g., characteristic tone of voice, dissociative attitude to a propositional content). We take our results to provide some support for the claim that the distinct tones of voice used in the two forms of irony may be linked to different mechanisms, with “regular” irony involving echoing alone and “parodic” irony involving both echoing and pretense. However, the lack of clear processing differences between ironic utterances produced with a deadpan compared with a parodic tone of voice could also indicate that tone of voice is potentially a less reliable ironic cue for children than cues such as context and facial expressions (cf. Bryant and Fox Tree, 2005; Deliens et al., 2018). Another possibility, as one reviewer pointed out, might be that from an acoustic point of view the stimuli with deadpan intonation were not sufficiently different from those with a parodic intonation to allow the children to discriminate between them (although adults clearly did).

All in all, the effects of both tone of voice and norm type were only evident for certain age groups, certain time windows or certain types of data. This is why these findings need to be interpreted with caution and require replication in future studies.

### A “Literal” Bias in Interpretation?

One clear advantage of including an online measure of eye-tracking in our study of irony comprehension was that it allowed us to explore what is going on when children misinterpret irony. Do they interpret ironical utterances as if they were literal, positive utterances? For instance, in (1) above, would they actually think that the speaker is praising the addressee for helping her, even if the context should clearly suggest otherwise? In the offline picture selection task, we expected young children to choose mainly literal interpretations, a prediction that was borne out by the data. In fact, the below-chance performance of 3-year-olds in the picture selection task suggests that they have a strong bias to interpret ironical utterances literally, similar to how they interpret cases of literal praise. The gaze data, however, provide a more refined picture. As we have seen, ironical utterances led to significantly more looks to the angry compared with the happy emoticon during most of the utterance. This shows that these young children are not naively mistaking irony for sincere praise, but that they are sensitive to cues such as context and/or tone of voice, which are indications that the speaker is likely to have a negative attitude. However, once 3- to 5-year-olds have analyzed the complete utterance, the gaze pattern for ironical utterances resembles that of literal praise utterances. This suggests that at this age, the semantic content of the ironical utterance is prioritized over other types of information such as context and tone of voice.

Our results have implications for the discussion of a possible “literal stage” in pragmatic development. First, they do not mesh well with the claim that children process utterances literally, piece-by-piece, even if it does not make sense in the context (cf. Levorato and Cacciari, 2002). If this were true, we should expect children to perform consistently below chance on the picture selection measure in our irony comprehension task. While this was the case for the 3-year-olds in our study, their eye-tracking behavior clearly suggests that they take context and/or tone of voice into account when processing ironical utterances, although they ultimately end up with a “wrong,” literal interpretation. Second, our results corroborate evidence from other tasks combining picture selection and eye-tracking to test figurative language comprehension (e.g., metonymy; Köder and Falkum, 2020). These show that the literal preference that young children tend to show on offline measures such as picture selection is discontinuous with their more “pragmatically appropriate” gaze behavior on eye-tracking measures, and that the latter might be more revealing not only of their actual pragmatic processing of the utterance but also of their pragmatic competence more generally.

One possible explanation for the 3- to 5-year-olds’ poor performance on the picture selection task could be the rather heavy demands that irony comprehension places on their still developing perspective-taking abilities, and that children in this age group are simply unable to grasp that a speaker is tacitly dissociating herself from a thought she attributes to someone else. While this is likely to be one side of the explanation, we take our results to suggest that the situation is more nuanced than this, with young children being sensitive to some of irony’s features well before they show any understanding on offline measures. Also, as we have discussed above, irony has an affinity with other pragmatic phenomena of which children show an early appreciation, such as pretense, jokes, and humor, some of which might also – in certain cases, at least – require some rather complex perspective-taking. More research is needed to pin down the contribution of several different factors – including children’s perspective-taking abilities – in the development of irony comprehension.

## Conclusion

In this study, we investigated the comprehension of irony in children aged 3–8 years. We took as our starting point the relevance-theoretic account of verbal irony, focusing on two of irony’s distinctive features as defined by this theory: (i) the normative bias and (ii) the characteristic tone of voice. We manipulated these two factors, namely, the violation of different types of norms and the use of different tones of voice, to see how they affected children’s processing and interpretation of irony. While the type of norm violation affected 4- to 5-year-olds’ offline understanding of irony, with a better performance on moral norm violations, tone of voice did not have a significant effect on children’s online gaze behavior, although the parodic tone of voice tended to lead to more looks to the angry compared with the happy emoticon at the offset of the ironical utterance.

All our experimental items were cases of “echoic” irony in the sense of Wilson and Sperber (2012), where the speaker is tacitly dissociating herself from an attributed thought (belief, norm-based expectation). Our results show that the understanding of irony in this sense can be detected on explicit measures around age 6 – around the same age as second-order perspective-taking abilities emerge – but that a sensitivity to several of irony’s features can be seen in both offline and online measures several years earlier. With links between developmental studies and theoretical accounts of irony understanding (in adults) hitherto being largely absent, our study can be seen as one step toward connecting these two strands of research and thereby contributing to building a more coherent account of the development of irony understanding.

Finally, our study provides novel input to the debate on the existence of a so-called literal stage in pragmatic development, in particular regarding 3-year-olds’ differential performance on the offline and online measures of irony understanding, suggesting that they are not naively mistaking ironical utterances for “ordinary” literal ones.

## Data Availability Statement

The raw data supporting the conclusions of this article will be made available by the authors, without undue reservation.

## Ethics Statement

The studies involving human participants were reviewed and approved by NSD - Norwegian Centre for Research Data. Written informed consent to participate in this study was provided by the participants’ legal guardian/next of kin.

## Author Contributions

FK and IF designed and conducted the study in collaboration. FK wrote the methodology and results sections, and IF wrote the introduction and discussion sections. Both the authors contributed to the article and approved the submitted version.

### Conflict of Interest

The authors declare that the research was conducted in the absence of any commercial or financial relationships that could be construed as a potential conflict of interest.

## Footnotes

^1^Note that we did not include so-called ironic compliments, i.e., ironically praising someone (e.g., “Bad job!” uttered when someone succeeded), since irony is typically used to express a dismissive attitude (Wilson and Sperber, 2012).

^2^Note that when utterance type by participant random slopes were included, the model failed to converge.

^3^Model: Norm_type_combined + (1|Participant), Note that the model failed to converge when Norm_type_combined by participant random slopes were included.

^4^Note that random smooths for participants and items did not improve the model fit significantly.

^5^The values on when differences between conditions are significant were calculated using the “plot_diff” function from the R package “itsadug” (van Rij et al., 2017).

^6^Model: Utterance_type + (1 | Participant) + (1 | Stimulus), data: adults, irony data, time window: 3,300–3,800 ms.

^7^Model: Utterance_type + (1 | Participant), data: children aged 3–8, irony data, time window: 3,300–3,800 ms.
